# *Eye*-Light on Age-Related Macular Degeneration: Targeting Nrf2-Pathway as a Novel Therapeutic Strategy for Retinal Pigment Epithelium

**DOI:** 10.3389/fphar.2020.00844

**Published:** 2020-06-05

**Authors:** Michele Catanzaro, Cristina Lanni, Filippo Basagni, Michela Rosini, Stefano Govoni, Marialaura Amadio

**Affiliations:** ^1^ Section of Pharmacology, Department of Drug Sciences, University of Pavia, Pavia, Italy; ^2^ Department of Pharmacy and Biotechnology, University of Bologna, Bologna, Italy

**Keywords:** Nrf2, age-related macular degeneration (AMD), retinal pigment epithelium (RPE), oxidative stress, pharmacological modulation, cytoprotection, HO1, p62

## Abstract

Age-related macular degeneration (AMD) is a common disease with a multifactorial aetiology, still lacking effective and curative therapies. Among the early events triggering AMD is the deterioration of the retinal pigment epithelium (RPE), whose fundamental functions assure good health of the retina. RPE is physiologically exposed to high levels of oxidative stress during its lifespan; thus, the integrity and well-functioning of its antioxidant systems are crucial to maintain RPE homeostasis. Among these defensive systems, the Nrf2-pathway plays a primary role. Literature evidence suggests that, in aged and especially in AMD RPE, there is an imbalance between the increased pro-oxidant stress, and the impaired endogenous detoxifying systems, finally reverberating on RPE functions and survival. In this *in vitro* study on wild type (WT) and Nrf2-silenced (siNrf2) ARPE-19 cells exposed to various AMD-related *noxae* (H_2_O_2_, 4-HNE, MG132 + Bafilomycin), we show that the Nrf2-pathway activation is a physiological protective stress response, leading downstream to an up-regulation of the Nrf2-targets HO1 and p62, and that a Nrf2 impairment predisposes the cells to a higher vulnerability to stress. In search of new pharmacologically active compounds potentially useful for AMD, four nature-inspired hybrids (NIH) were individually characterized as Nrf2 activators, and their pharmacological activity was investigated in ARPE-19 cells. The Nrf2 activator dimethyl-fumarate (DMF; 10 μM) was used as a positive control. Three out of the four tested NIH (5 μM) display both direct and indirect antioxidant properties, in addition to cytoprotective effects in ARPE-19 cells under pro-oxidant stimuli. The observed pro-survival effects require the presence of Nrf2, with the exception of the lead compound NIH1, able to exert a still significant, albeit lower, protection even in siNrf2 cells, supporting the concept of the existence of both Nrf2-dependent and independent pathways mediating pro-survival effects. In conclusion, by using some pharmacological tools as well as a reference compound, we dissected the role of the Nrf2-pathway in ARPE-19 stress response, suggesting that the Nrf2 induction represents an efficient defensive strategy to prevent the stress-induced damage.

## Introduction

Age-related macular degeneration (AMD) is one of the most common neurodegenerative diseases and leading causes of irreversible blindness in the elderly worldwide (Lim et al., 2012). AMD is characterized by a progressive loss of central vision due to degenerative and vascular changes in the macula, the retinal region responsible for fine and color vision (Jager et al., 2008). AMD is classified in dry (~90%) and wet (~10%) AMD forms, in early and late stages (Lim et al., 2012). Presently, no cure is available for dry AMD. In the wet form, monthly intravitreous injections of anti-VEGF drugs are used to contrast the neoangiogenesis, though they can only delay the symptoms and present several limits (Amadio et al., 2016).

The degeneration of retinal pigment epithelium (RPE), fundamental for photoreceptor homeostasis and, in general, for retina health, is among the earliest factors triggering AMD pathology (Bhutto and Lutty, 2012). Compared with normal RPE, AMD RPE presents increased susceptibility to oxidative stress, autophagy impairment and higher levels of reactive oxygen species (ROS) under stress conditions (Golestaneh et al., 2018). The most comprehensive transcription system used by RPE to neutralize oxidative stress and maintain cellular homeostasis is the Keap1-Nrf2-ARE pathway (Sachdeva et al., 2014; Lambros and Plafker, 2016). The nuclear factor E2-related factor 2 (Nrf2) is a transcription factor that is activated/upregulated under oxidative stress. In basal conditions, in the cytosol Nrf2 is anchored by Kelch-like ECH-associated protein 1 (Keap1), which mediates Nrf2 proteasomal degradation, maintaining Nrf2 at a low level (McMahon et al., 2003). Upon oxidative stress, Keap1 undergoes a conformational change and dissociates from Nrf2, that is free to translocate to the nucleus, where it binds to the antioxidant response element (ARE) in the promoter of target genes, thus initiating their transcription (Motohashi and Yamamoto, 2004). Nrf2 activation has been shown to protect against oxidative stress, protein deposition, inflammation (Zhang et al., 2015; Pajares et al., 2016). Since oxidative stress is one of the main factors contributing to the AMD pathogenesis, and literature evidence suggests that Nrf2-signalling pathway is compromised in AMD-like animal models (Sachdeva et al., 2014; Batliwala et al., 2017), we evaluated the relevance of Nrf2 in RPE under adverse conditions.

In particular, in wild type and Nrf2-deficient ARPE-19 cells, we studied the effects of the following pro-oxidant AMD-related *noxae*: H_2_O_2_, 4-hydroxynonenal (4-HNE), MG132 + Bafilomycin. H_2_O_2_ is a strong oxidant leading to an immediate ROS production, widely used in *in vitro* study on RPE cells (Zhu et al., 2017; Hu et al., 2019; Zhao et al., 2019), that are physiologically subjected to elevated ROS levels due to their high metabolism and functions (Strauss, 2005). 4-HNE is a product of lipid peroxidation accumulating in AMD retina (Ethen et al., 2007); it is pro-oxidant and toxic for RPE (Kaarniranta et al., 2005; Kaemmerer et al., 2007; Chen et al., 2009), but its effects on Nrf2-pathway have been not fully elucidated. MG132 + Bafilomycin co-stimulus inhibits autophagy in RPE (Viiri et al., 2010; Viiri et al., 2013), leading to accumulation of protein aggregates, a condition that predisposes to a more oxidant intracellular environment and dysfunction of RPE (Hyttinen et al., 2014; Ferrington et al., 2016). In stressed ARPE-19 cells, among Nrf2-responsive genes, we studied the modulation of both *Heme Oxygenase 1* (*HO1*) and *p62/sequestosome 1* (*p62/SQSTM1*), in virtue of their acknowledged functions in the maintenance and survival of RPE in the adult retina (Loboda et al., 2016; Wang et al., 2016). In particular, HO1 is a detoxifying enzyme with a role in retina physiopathology and ocular diseases (Zhao et al., 2012), while p62 is a key regulator of proteostasis in RPE (Kaarniranta et al., 2017), and it mediates Keap1 degradation, contributing to Nrf2 activation in a positive feedback loop (Jain et al., 2010; Jiang et al., 2015).

With the aim to find new pharmacological tools potentially useful in AMD, we also tested a small set of nature-inspired hybrids (here called NIH) carrying the hydroxycinnamoyl function recurring in polyphenols, and the allyl mercaptan moiety of garlic-derived organosulfur compounds. These compounds, previously characterized in other cellular models (Simoni et al., 2016; Simoni et al., 2017; Serafini et al., 2019), were herein studied for their antioxidant potential, their capability to activate Nrf2-pathway, and to promote protection in both wild type and Nrf2-deficient ARPE-19 cells under challenging stressful conditions.

## Materials and Methods

### Cell Culture, Reagents and Treatments

The human RPE cell line ARPE-19 was obtained from American Type Culture Collection. Cells were grown in a humidified 5% CO2 atmosphere at 37 °C in Dulbecco’s Modified Eagle Medium: F12 (1:1; Gibco, Invitrogen, Carlsbad, CA, USA), including 10% inactivated fetal bovine serum, 100 units/ml penicillin, 100 μg/ml streptomycin, and 2 mM L-glutamine (Merck KGaA, Darmstadt, Germany). The experiments were carried out on passages 15–20. Cells were exposed to either the solvent (0.05% DMSO), or H_2_O_2_ (300–500 μM; Merck KGaA), the lipid peroxidation product 4-HNE; 50–100 μM; Cayman, Ann Arbor, MI, USA), the proteasome inhibitor MG132 (5 μM; Calbiochem, San Diego, CA, USA), the Vacuolar H+-ATPase Inhibitor bafilomycin (50 nM; Merck KGaA). Nature-inspired hybrids (NIH) were synthesized according to previous procedures (Simoni et al., 2016; Simoni et al., 2017) and were >98% pure. Each NIH and dimethyl-fumarate (DMF, Merck KGaA) were dissolved in DMSO to obtain 10- and 20-mM stock solutions, respectively. NIH and DMF were diluted until 5 and 10 μM, respectively. Treatments were performed in triplicates, if not otherwise indicated.

### Silencing of Nrf2 Expression

A siRNA designed for the human *Nrf2* gene (Merck KGaA) was incubated for at least 24 h to obtain the siNrf2 ARPE-19 cell line. A commercial negative siRNA (siNEG, Merck KGaA) having no known homology with any gene was used as a negative control in preliminary experiments to confirm the specificity of the transient Nrf2 silencing. The siRNAs were transfected into ARPE-19 cells using the lipofectamin RNAiMAX transfection reagent (Invitrogen, Thermo Scientific, Waltham, MA, USA) following the manufacturer’s instructions; siRNA treatment was maintained throughout the experiments (up to 72 h). To confirm that Nrf2 expression was silenced, 4 h before the end of the experiment, the proteasome inhibitor MG132 (5 µM) was added to the medium of selected plates to block the degradation of Nrf2 protein, that was evaluated by Western blotting.

### Immunocytochemistry

ARPE-19 cells were seeded onto poly-L-lysin-coated coverslips for 24 h before exposure to either solvent, NIH or DMF, for 3 h. Immunocytochemistry was performed as previously described, with minor modifications (Marchesi et al., 2018). Briefly, cells were fixed in ethanol 70% at −20 °C, washed with phosphate-buffered saline (PBS), and permeabilized for 15 min with 0.01% Triton X-100 in PBS. Nonspecific binding sites were blocked at room temperature by incubation for 30 min with PBS containing 1% bovine serum albumin (BSA). Cells were then incubated for 1 h with a polyclonal antibody recognizing Nrf2 (NBP1-32822; Novus Biologicals, Centennial, CO, USA) diluted 1:50 in PBS/1% BSA solution. After a brief rinse with PBS solution, cells were incubated for 1 h with the Alexa Fluor 488-conjugated anti-rabbit secondary antibody (A27034; Invitrogen) diluted at 1:200 in PBS/1% BSA. Cells were rinsed in PBS, then incubated for 10 min with Hoechst solution to counterstain the nucleus. After rinse with PBS and distilled water, the cells were finally mounted up-side-down on a glass slide in a drop of Mowiol mounting medium (Merck KGaA). Cells were photographed with AxioCam MRc5 mounted on Zeiss Axioskop 40 microscopy.

### Cell Fractioning, Protein Extraction, and Western Blotting

ARPE-19 cells plated in either 35 or 100 mm dishes were subjected to treatments, then washed twice with cold PBS, scraped, and collected. For study on total homogenates, cells were lysed in an appropriate buffer (50 mM Tris-HCl pH 7.5, 150 mM NaCl, 5 mM EDTA, 0.5% Triton X-100, and protease inhibitors mix), and sonicated two times for 10 s. For Nrf2 translocation study, nuclear extracts were obtained by using the Nuclear Extract kit (Active Motif, Carlsbad, CA, USA) according to our previous publication (Viiri et al., 2013). Protein contents of total homogenates and nuclear fractions were determined by Bradford method (SERVA GmbH, Heidelberg, Germany) using BSA as a standard. Total lysate and nuclear fractions were diluted in 2X SDS protein gel loading solution and separated on 10% SDS-polyacrylamide gel electrophoresis, transferred into a nitrocellulose membrane, and processed following the standard procedures. The antibodies were diluted in 5% BSA in TBS-T Buffer (10 mM Tris-HCl, 100 mM NaCl, 0.1% Tween, pH 7.5) as follows: the anti-Nrf2 (NBP1-32822) and anti-HO1 (NBP1-31341) rabbit polyclonal antibodies (Novus Biologicals) at 1:1,000; the anti-p62 (sc-28359), anti-lamin A (sc-71481) (both by Santa Cruz Biotechnology, Inc., Dallas, TX, USA), anti-β-actin (612656; BD Biosciences, San Josè, CA, USA) mouse monoclonal antibodies at 1:1,000, 1:3,000, and 1:1,000, respectively. The horseradish peroxidase-conjugated secondary anti-mouse (A4416; Merck KGaA) and anti-rabbit (sc-2357; Santa Cruz Biotechnology, Inc.) antibodies were diluted in 5% BSA/TBS-T Buffer. The nitrocellulose membranes signals were detected by chemiluminescence. Experiments were performed in duplicate for each different cell preparation. As loading controls, β-actin was used for total homogenate, while lamin A for the rough nuclear fraction. Statistical analysis of the Western blotting data was performed on the densitometric values obtained by the Scion Image software (Scion Corporation).

### RNA Extraction, Retro-Transcription, and Real-Time Quantitative PCR

Total RNA was extracted from ARPE-19 cells by the Direct-zol RNA MiniPrep Kit (Zymo Research, Irvine, CA, USA) and subjected to reverse transcription by the QuantiTect Reverse Transcription Kit (Qiagen, Hilden, Germany) following standard procedures. Real-time quantitative PCR (RT-qPCR) amplifications were carried out using the QuantiTect SYBR Green PCR Kit (Qiagen) and the Lightcycler instrument (Roche, Basel, Switzerland), with the following primers:

Nrf2: 5′- TTCTGTTGCTCAGGTAGCCCC-3′ (upstream)
and 5′- TCAGTTTGGCTTCTGGACTTGG -3′ (downstream);
HO1: 5′- AGCAACAAAGTGCAAGATTCTGC -3′ (upstream)
and 5′- CAGCATGCCTGCATTCACATG -3′ (downstream);
GAPDH: 5′- CAGCAAGAGCACAAGAGGAAG-3′ (upstream)
and 5′- CAACTGTGAGGAGGGGAGATT -3′ (downstream).

GAPDH mRNA was the reference on which all the other values were normalized, due to its substantial stability in our experimental conditions as in most cases in literature. 2^−ΔΔCt^ method was used for quantification of mRNAs.

### Cell Viability Assays

ARPE-19 cells were plated 20,000/well in a 96-well plate, and cell viability was determined by either MTT (Merck KGaA) or PrestoBlue® (Invitrogen) assays. MTT assay was performed according to a published method (Amadio et al., 2008); absorbance was measured at 495 nm in a UV spectrophotometer and the results were expressed as a percentage of the absorbance of the samples in comparison to control. PrestoBlue® assay was used following manufacturer’s instruction. After treatments, cells were loaded for 10 min with PrestoBlue® reagent prior to assay readout. Fluorescence was measured by the Synergy HT multidetection microplate reader (BioTek, Winooski, VT, USA) with excitation and emission wavelengths of 530 and 590 nm, respectively. The results were expressed as a percentage of the fluorescence of the samples in comparison to control.

### Measurement of Reactive Oxygen Species

Measurement of intracellular reactive oxygen species (ROS) was performed by 2′,7′-Dichlorofluorescin diacetate (DCFH-DA) assay (Merck KGaA) following manufacturer’s instruction. Two different experimental settings were followed, as described in the relative figure legends. In each setting, at the end of the H_2_O_2_ treatments, cells were detached by trypsin, counted, incubated with 25 μM DCFH-DA for 45 min, and centrifuged (1,200×*g* for 5 min at 37 °C), to remove the DCFH-DA. The cells were re-suspended in medium without FBS, plated in black-bottom 96-well plate, and the 2′,7′‐dichlorofluorescin (DCF) was measured (λ_ex_ = 485 nm, λ_em_ = 530 nm) by Synergy HT multidetection microplate reader (BioTek).

### Heme Oxygenase Activity

Cells cultured in 100 mm diameter petri dishes were collected after incubation with either solvent, NIH1, NIH4 (5 μM for 6 h), or hemin (10 μM for 4 h), a well-known HO1 inducer (Amadio et al., 2014). Heme oxygenase activity was assessed according to a published method with minor modifications (Foresti et al., 2015). Briefly, samples were incubated with the substrate hemin, biliverdin reductase, NADPH, glucose-6-phosphate (G6P), G6P dehydrogenase (Merck KGaA), and other reagents to sustain heme oxygenase activity. The assay is based on the spectrophotometric determination of bilirubin as the final product of a reaction where hemin is transformed by heme oxygenase to biliverdin, which is in turn converted by biliverdin reductase to bilirubin. The reaction was allowed to proceed for 1 h at 37 °C in the dark and was stopped by addition of chloroform to extract the bilirubin formed. The extracted bilirubin was measured spectrophotometrically (wavelengths of 464 and 530 nm) and calculated in picomoles bilirubin/mg ARPE-19 cell protein/h.

### NIH Stability Assay

NIH1 was dissolved in DMSO to obtain a stock solution 10 mM. Samples of this stock solution were diluted until 1 mM in cell-free complete culture medium and left incubating at 37 °C for up to three days. At selected time points (0, 24, 48, and 72 h) samples were collected, diluted at 0.1 mM with mobile phase ACN/H2O 40:60, and analyzed through HPLC reversed-phase conditions on a Phenomenex Jupiter C18 (150 × 4.6 mm I.D.) column, UV detection at λ = 302 nm and a flow rate of 1 ml/min. Analyses were performed on a liquid chromatograph model PU 2089 PLUS equipped with a 20 µl loop valve and linked to MD 2010 Plus UV detector (Jasco Europe, Lecco, Italy). Areas of NIH1 peak, identified by co-injection, were analyzed and their percentage reductions vs time were reported in the graph.

### Statistical Analysis

For the statistical analyses the GraphPad InStat program (GraphPad software, San Diego, CA, USA) was used. Data were subjected to the analysis of variance (either one-way or two-way ANOVA) followed, when significant, by an appropriate *post hoc* comparison test, as specifically indicated. Differences were considered statistically significant when p <0.05.

## Results

### The Nrf2-Deficit Reverberates on the Cell Viability in ARPE-19 Cells Under Stress

To evaluate the efficiency rate of our Nrf2-silencing RNA, we first measured, by both RT-qPCR and Western blotting, the Nrf2 expression in wild type (WT), negative-siRNA (siNEG), and Nrf2-silenced (siNrf2) ARPE-19 cells, confirming in the latter ones a specific and marked decrease of both Nrf2 mRNA and protein content (**Supplementary Figure 1**). No difference in Nrf2 expression between WT and siNEG cells was found.

To determine whether the Nrf2 impairment affects the susceptibility of ARPE-19 to stress in term of cell viability, according to both our previous experience and new explorative experiments, we preliminary selected the best conditions (time/concentration) for each stress inducing a significant mortality in WT ARPE-19 cells.

**Figure 1:**
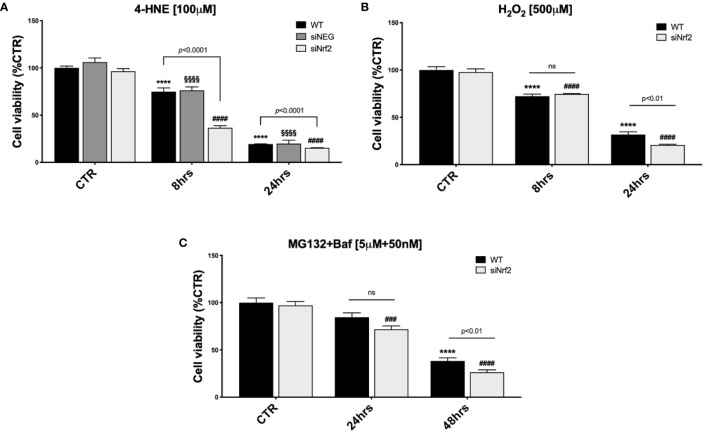
The Nrf2-impaired ARPE-19 cells are more susceptible to stress than their WT counterpart. Cell viability was assessed by a fluorometric assay (PrestoBlue®) and results are expressed as mean of percentages ± SEM. **(A)** WT, siNEG, and siNrf2 ARPE-19 cells were treated with either solvent (DMSO, CTR) or 4-HNE [100 μM] for 8 and 24 h. Dunnett’s multiple comparisons test; ****p < 0.0001 versus WT CTR; ^§§§§^p < 0.0001 versus siNEG CTR; and ^####^p < 0.0001 versus siNrf2 CTR; ns = non significant; n = 5–8. **(B)** WT and siNrf2 ARPE-19 cells were treated with either solvent (DMSO, CTR) or H_2_O_2_ [500 μM] for 8 and 24 h. Sidak’s multiple comparisons test; ****p = 0.0001 versus WT CTR; ^####^p < 0.0001 versus siNrf2 CTR; n = 6–8. **(C)** WT and siNrf2 ARPE-19 cells were treated with either solvent (DMSO, CTR) or MG132 + Bafilomycin [5 μM + 50 nM] for 24 and 48 h. Sidak’s multiple comparisons test; ****p < 0.0001 versus WT CTR; ^###^p < 0.001 and ^####^p < 0.0001 versus siNrf2 CTR; ns = non significant; n = 5–9.

WT ARPE-19 cells were either non-stressed, or exposed to 30, 50, or 100 µM 4-HNE, for 8 and 24 h, and analyzed by a cell viability assay (**Supplementary Figure 2A**). Upon 8 h exposure to either 30 or 50 µM 4-HNE, no reduction in the cell viability was detected, while a significant mortality was observed following 100 µM 4-HNE. At 24 h, we found a dose–response proportional decrease in the viability of 4-HNE-stressed ARPE-19 cells. Therefore, WT, siNEG, and siNrf2 ARPE-19 cells were either non-stressed, or exposed to 100 µM 4-HNE, for 8 and 24 h, and analyzed for cell viability (**Figure 1A**). In basal conditions, there is no difference in the viability among the three cell lines. At both 8 and 24 h under 4-HNE, siNrf2 cells show a lower survival than WT cells. Under stress, siNEG cells show a mortality fully comparable to WT cells; for this reason, we performed the following experiments only in WT and siNrf2 cells.

We also analyzed the viability of WT ARPE-19 cells that were either non-stressed, or exposed to 100, 300, or 500 µM H_2_O_2_, for 8 and 24 h (**Supplementary Figure 2B**), and then we selected 500 µM H_2_O_2_ for 24 h as the best condition for our purpose. Both WT and siNrf2 ARPE-19 cells were either non-stressed, or exposed to 500 µM H_2_O_2_, for 8 and 24 h, and analyzed for viability; we found that siNrf2 cells show a lower survival than WT cells at 24 h (**Figure 1B**).

According to our previous experience with autophagy inhibitors in ARPE-19 cells (Viiri et al., 2013), that require longer times to display cytotoxicity, we evaluated the impact of MG132 + Bafilomycin (MG132+Baf; 5 µM + 50 nM) co-treatment, for 24 and 48 h, in both WT and siNrf2 cells, and found a more consistent mortality in the latter ones at 48 h (**Figure 1C**).

Overall, these results indicate that the viability of WT and siNrf2 cells is fully comparable in normal conditions, while in prolonged adverse conditions the Nrf2 impairment predisposes the ARPE-19 cells to a higher stress-induced mortality.

### Different Stress Stimuli Activate Nrf2-Pathway in Wild Type ARPE-19 Cells

**Figure 2:**
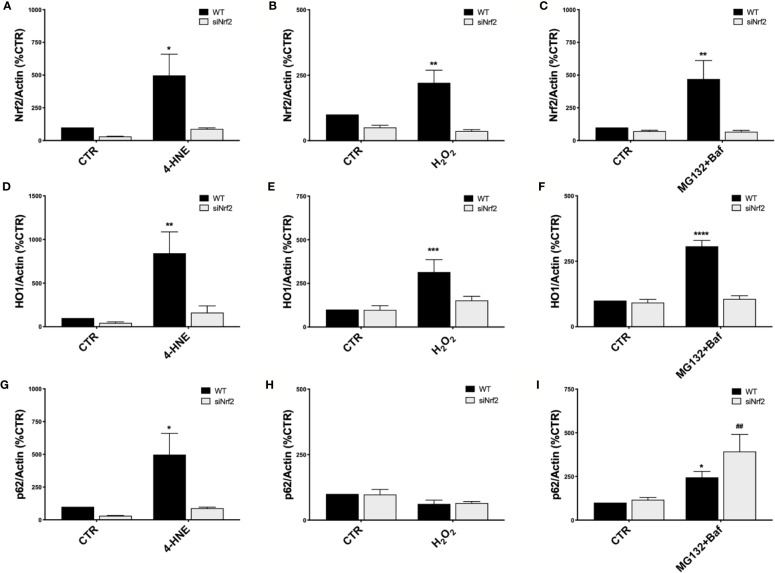
Nrf2-pathway activation in wild type ARPE-19 cells under different stress conditions. Both WT and siNrf2 ARPE-19 cell lines were treated for 6 h with either solvent (DMSO, CTR), 4-HNE [50 μM] **(A**, **D**, **G)**, H_2_O_2_ [300 μM] **(B**, **E**, **H)**, or MG132 + Bafilomycin [5 μM + 50 nM] **(C**, **F**, **I)**. Total cellular homogenates were examined by Western blot using an antibody against Nrf2 **(A**–**C)**, HO1 **(D**–**F)**, and p62 **(G**–**I)**; Actin content was used as a loading control to normalize the data. Results are expressed as percentage means ± SEM. Sidak’s multiple comparisons test; *p < 0.05, **p < 0.01, ***p < 0.001, and ****p < 0.0001 versus WT CTR; ^##^p < 0.01 versus siNrf2 CTR; n = 4–6.

To study the Nrf2-pathway activation under pro-oxidant injury without causing cell mortality, both WT and siNrf2 ARPE-19 cells were either non-stressed, or exposed to 4-HNE (50 µM), H_2_O_2_ (300 µM), and MG132+Baf (5 µM + 50 nM) co-treatment for 6 h. We first evaluated Nrf2 protein levels in total cellular homogenates by Western blotting, finding that each of these stressful conditions up-regulates Nrf2 protein expression in WT but not in siNrf2 cells, as expected (**Figures 2A–C**).

In order to highlight potential specificities in the stress-induced activation of Nrf2-targets, we then evaluated downstream the expression of HO1 and p62 protein levels in the same cellular total homogenates by Western blotting. We found that HO1 protein content is increased in WT cells in all the noxious conditions examined; no significant change in HO1 levels was detected in the stressed siNrf2 cells, suggesting the strong dependence of HO1 expression on Nrf2 (**Figures 2D–F**). The p62 protein level varies in function of the stress condition: it is not changed in H_2_O_2_-treated WT cells, while it is increased in WT cells exposed to either 4-HNE or MG132+Baf co-treatment (**Figures 2G–I**). In siNrf2 cells exposed to either 4-HNE or H_2_O_2_, there are no alterations in p62 protein amount. Instead, a marked increase of p62 level occurs in siNrf2 cells under MG132+Baf, in accordance to the degradation of p62 via autophagy which is indeed blocked by this co-treatment. The increase of p62 protein level triggered by MG132+Baf seems even higher in siNrf2 than in WT cells, suggesting that siNrf2 cells are featured by a less efficient autophagy flux that may be further compromised in the presence of autophagy inhibitors.

### NIH Are Well-Tolerated and Activate Nrf2-Pathway in ARPE-19 Cells

In order to find new pharmacological tools potentially useful in AMD, we tested some nature-inspired hybrids (NIH1-4; **Supplementary Figure 3A**) for their capability to activate Nrf2-pathway in ARPE-19 cells. Previous *in vitro* studies showed that NIH allow Nrf2 activation when at least a (pro)electrophilic feature, such as the catechol moiety or the α,β-unsatured carbonyl group, is present (Simoni et al., 2017; Serafini et al., 2019). First, preliminary experiments on the lead compound NIH1 were performed to evaluate its capability to induce Nrf2 nuclear translocation in ARPE-19 cells. According to our evidence in another cellular line (Serafini et al., 2019), ARPE-19 cells were exposed for 3 h to either the solvent or lead compound NIH1 at two concentrations (0.5 and 5 µM), and Nrf2 protein content within the nuclear fractions was evaluated by Western blotting experiments. We found that ARPE-19 cells exposed to 5 µM NIH1 display significantly higher Nrf2 nuclear levels compared to control cells (control: 100.0% ± 12.6; 0.5 µM NIH1: 110.0% ± 8.2; 5 µM NIH1: 166.7%* ± 11.4; values expressed as mean percentage ± SEM; Dunnett’s multiple comparisons test, *p <0.05 vs control; n = 4). Consistently, 5 µM concentration was selected and used for each NIH in the following experiments. As a positive control of Nrf2 activation we used dimethyl-fumarate (DMF, 10 μM).

**Figure 3:**
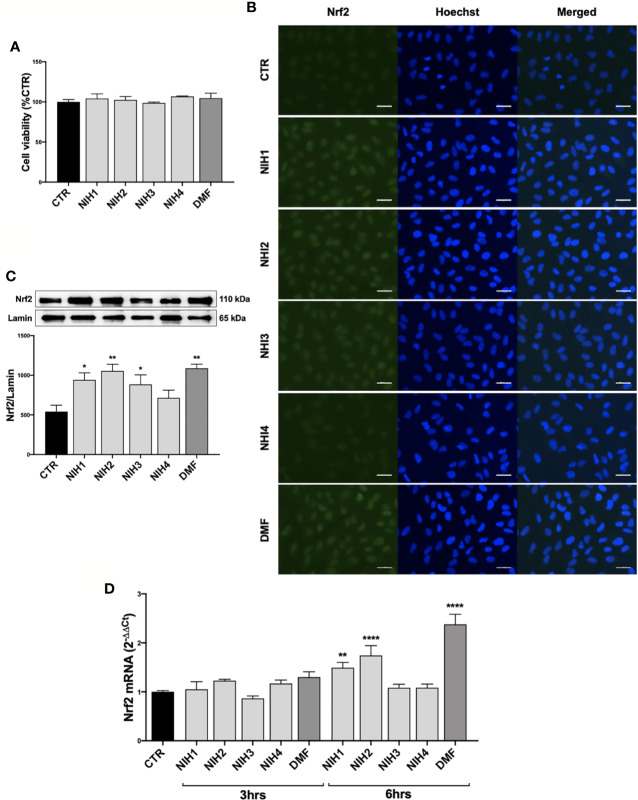
Pharmacological activation of Nrf2 protein by NIH in ARPE-19 cells. **(A)** Tolerability of NIH in ARPE-19 cells. ARPE-19 cells were treated for 48 h with solvent (DMSO, CTR), NIH1, NIH2, NIH3 and NIH4 [5 μM], or DMF [10 μM], and their viability was assessed by MTT assay. Results are expressed as mean of percentages ± SEM (CTR: 100%); Dunnett’s multiple comparisons test; no statistical differences versus CTR; n = 3–5. **(B**–**C)** Evaluation of NIH-induced Nrf2 nuclear translocation. ARPE-19 cells were treated for 3 h with either solvent (DMSO, CTR), NIH1, NIH2, NIH3, NIH4 [5 μM], or DMF [10 μM].**(B)** Immunocytochemistry for Nrf2 protein localization was performed by using a green fluorescent-conjugated secondary antibody. Nuclei (blue) were stained by Hoechst. Scale bar: 20 μm. **(C)** Nuclear fraction was isolated and examined by Western blot using an antibody against Nrf2. Lamin A content was used as a loading control to normalize the data. Results are expressed as means of Nrf2/Lamin A (ratio × 1000) ± SEM. Dunnett’s multiple comparisons test; *p<0.05 and **p<0.01 versus CTR; n=5. **(D)** Study of *Nrf2* mRNA expression following NIH treatment. Total *Nrf*2 mRNA levels were measured by RT-qPCR in ARPE-19 cells exposed to either solvent (DMSO, CTR), NIH1, NIH2, NIH3, NIH4 [5 μM], or DMF [10 μM] for 3 and 6 h. *GAPDH* mRNA content was used as a housekeeping to normalize the data. Results are expressed as means of 2^−ΔΔCt^ ± SEM. Dunnett’s multiple comparisons test; **p <0.01 and ****p <0.0001 versus CTR; n = 3.

We determined the tolerability of NIH1, NIH2, NIH3, and NIH4 by MTT assay, finding that 48 h exposure does not affect the ARPE-19 cell viability (**Figure 3A**). A stability test on NIH1 performed in the complete culture medium at 24, 48, and 72 h, indicates that, compared to time 0, NIH1 concentration is reduced to 74.18% ± 2.80, 46.26% ± 1.56, and 18.17% ± 0.6, respectively (**Supplementary Figure 3B**).

We then evaluated the capability of hybrids to activate Nrf2 in ARPE-19 cells. Following 3 h exposure, the NIH1, NIH2, NIH3, having in their structure the chemical group(s) responsible for the Nrf2-pathway activation (Simoni et al., 2016) (**Supplementary Figure 3A**), induce an increase of Nrf2 content within the nucleus, as shown by both immunocytochemistry (**Figure 3B**) and Western blotting experiments (**Figure 3C**). Immunocytochemistry revealed a general increment of Nrf2-immunostaining in the whole ARPE-19 cells exposed to either NIH1, NIH2, or NIH3. To confirm a possible up-regulation of Nrf2 expression upon these hybrids, by RT-qPCR we measured total Nrf2 mRNA levels at both 3 and 6 h, finding a statistically significant increase after 6 h treatment with NIH1 and NIH2 (**Figure 3D**), consistent with a self-sustaining positive feedback of Nrf2 itself. On the contrary, the fact that NIH3 does not increase Nrf2 mRNA levels after 3 and 6 h may be ascribed to our previous observation that some of the investigated compounds may affect the Nrf2-pathway following different temporal kinetics (Serafini et al., 2019). DMF is well tolerated after 48 h exposure; it induces Nrf2 nuclear translocation at 3 hrs, and up-regulates Nrf2 mRNA expression at 6 h (**Figure 3**). NIH4, lacking the Nrf2-activating functional groups (**Supplementary Figure 3A**), instead displays effects not different from the solvent (**Figure 3**); therefore, NIH4 will be mentioned as “inactive” NIH since now.

### The Nrf2-Target *HO1* Gene Can Be Differently Induced by the Active NIH in ARPE-19 Cells

**Figure 4:**
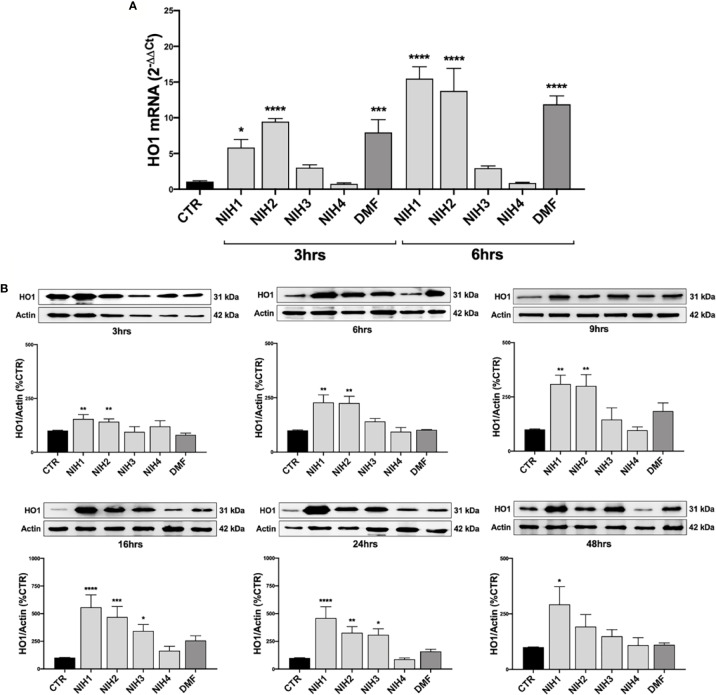
The NIH-mediated increase of HO1 expression vary among molecules. **(A)** Total *HO1* mRNA levels were measured by RT-qPCR in ARPE-19 cells exposed to either solvent (DMSO, CTR), NIH1, NIH2, NIH3 and NIH4 [5 μM], or DMF [10 μM], for 3 and 6 h. *GAPDH* mRNA content was used as a housekeeping to normalize the data. Results are expressed as means of 2^−ΔΔCt^ ± SEM. Dunnett’s multiple comparisons test; *p <0.05, ***p <0.001 and ****p <0.0001 versus CTR; n = 3. **(B)** ARPE-19 cells were exposed to either solvent (DMSO, CTR), NIH1, NIH2, NIH3, NIH4 [5 μM], or DMF [10 μM], for increasing times (up to 48 h), and total homogenates were examined by Western blot using an antibody against HO1. Actin was used as a loading control to normalize the data. Results are expressed as mean percentages ± SEM. Dunnett’s multiple comparisons test; *p <0.05, **p <0.01; ***p <0.001 and ****p <0.0001 versus CTR; n = 5.

Because of the above results and tight dependence on Nrf2, we selected *HO1* gene to evaluate the effects of NIH downstream *Nrf2* in WT ARPE-19 cells. In line with data on *Nrf2* mRNA levels, we found that total HO1 mRNA levels are significantly higher after 3 and 6 h exposure to NIH1 and NIH2 (**Figure 4A**). NIH1, NIH2, and NIH3, up-regulate total HO1 protein expression, although with extents and/or time-courses that vary among molecules (**Figure 4B**). In particular, after 3 h treatment, both NIH1 and NIH2 lead to a significant increase of HO1 protein content, that is maintained for the longer times here considered, and is still sustained at 24 h. The NIH1-mediated increase of HO1 protein is relevant even after 48 h treatment. For NIH3, we found an increase of total HO1 protein after 16 and 24 h (**Figure 4B**). DMF induces an up-regulation of *HO1* mRNA at both 3 and 6 h (**Figure 4A**); we also observed a trend to increase of HO1 protein levels upon 9, 16, 24 h DMF treatment, but without statistical significance in the overall group (**Figure 4B**). However, when comparing DMF and control alone (by an Unpaired t test), in DMF-treated cells HO1 protein levels are significantly higher than control after 9 h (+84.3% ± 37.7; n = 3–5, p <0.05), 16 h (+157.1% ± 43.0; n = 5, p <0.001), and 24 h (+58.9% ± 19.8; n = 5, p <0.01). No effects on *HO1* mRNA and protein expression are observed for the inactive NIH4 (**Figure 4**).

### NIH1, NIH2, NIH3 Have Both Direct and Indirect Antioxidant Properties in ARPE-19 Cells

**Figure 5:**
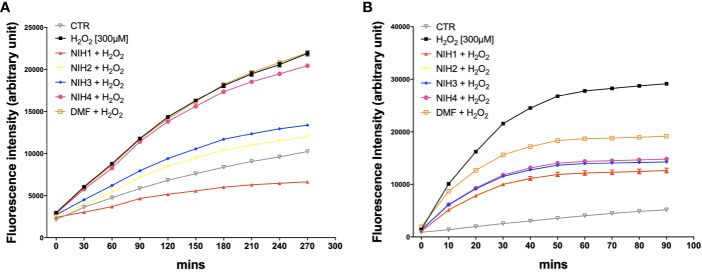
NIH are endowed of anti-oxidant properties. **(A)** ARPE-19 cells were exposed to H_2_O_2_ [300 μM] ± NIH1, NIH2, NIH3 and NIH4 [5 μM], or DMF [10 μM] for 4.5 h. Control cells were exposed only to solvent (DMSO). ROS levels were measured every 30 min by the fluorometric 2′,7′-Dichlorofluorescin diacetate (DCF-DA) assay. Results are expressed as mean ± SEM. Fluorescence intensity for the NIH1, NIH2, NIH3, is significantly different from H_2_O_2_ at any time starting from 30 min with p <0.001. Dunnett’s multiple comparisons test versus H_2_O_2_; n = 4. **(B)** ARPE-19 cells were pre-treated for 24 h with either solvent (DMSO, CTR), NIH1, NIH2, NIH3, NIH4 [5 μM], or DMF [10 μM], and then exposed to H_2_O_2_ [300 μM] for 1.5 h. ROS levels were measured every 10 min by the DCF-DA assay. Results are expressed as mean ± SEM. Fluorescence intensity for all the NIH and DMF is significant at any time starting from 10 min with p <0.0001. Dunnett’s multiple comparisons test versus H_2_O_2_; n = 5.

We then evaluated the potential direct and indirect antioxidant properties of each NIH in H_2_O_2_-exposed ARPE-19 cells. To determine the direct antioxidant activity, ARPE-19 cells were either non-stressed, or exposed to 300 µM H_2_O_2_ with/without NIH, or DMF. By the DCF-DA assay, we measured ROS levels every 30 min during the following 4.5 h, finding that all the active NIH display ROS-scavenging capability, which is the highest for NIH1, followed by NIH2 and NIH3, respectively (**Figure 5A**). The ROS levels detected in ARPE-19 cells exposed to H_2_O_2_ plus NIH4 are slightly lower, while in DMF-treated cells they are comparable to those of H_2_O_2_-treated cells.

In order to evaluate whether the active NIH may provide protection from H_2_O_2_ not only by acting directly, as radical scavengers, but also because of the ability to induce an antioxidant cellular response, the ARPE-19 cells were pre-treated for 24 h with NIH, and then exposed to 300 µM H_2_O_2_. The ROS production was detected every 10 min for 1.5 h. We found that, compared to those in stressed cells, the ROS levels are progressively lower in cells pre-treated with NIH1, NIH2, followed by NIH3, NIH4 and, to a less extent, DMF (**Figure 5B**). The antioxidant effect observed here in NIH4-treated cells should be further studied.

According on these results, by selecting the most active among our hybrids, we evaluated the impact of NIH1 on the HO enzymatic activity at 6 h, and found a significant increase of the HO metabolic product biliverdin in NIH1-treated ARPE-19 cells compared to control and NIH4-treated cells (**Supplementary Figure 4**).

### NIH1 Provides Protection From Stress Stimuli in Both WT and Nrf2-Silenced ARPE-19 Cells

**Figure 6:**
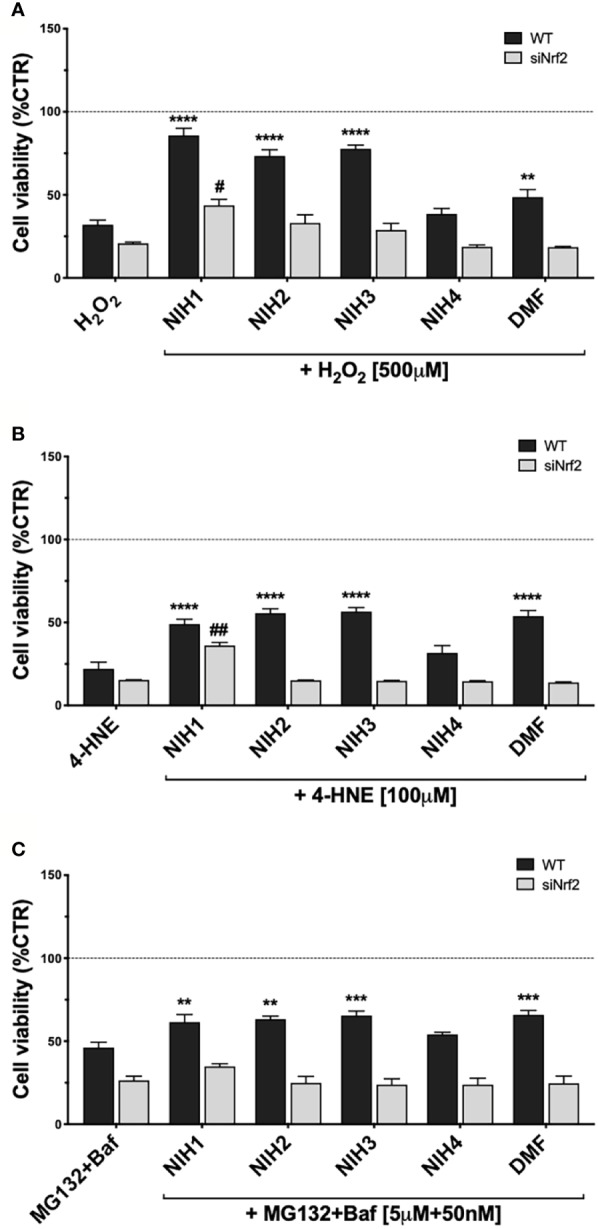
Study of NIH-mediated protection in WT and siNrf2 ARPE-19 cells under different stress. Cell viability was assessed by a fluorometric assay (PrestoBlue ®) in WT and siNrf2 ARPE-19 cells pre-treated for 24 h with either solvent (DMSO, CTR), NIH1, NIH2, NIH3, NIH4 [5 μM], or DMF [10 μM], and then exposed to either H_2_O_2_ [500 μM, for 24 h] **(A)**, 4-HNE [100 μM, for 24 h] **(B)**, or MG132 + Bafilomycin [5 μM + 50 nM, for 48 h] **(C)**. Results are expressed as mean of percentages ± SEM in comparison with control (dot line, 100%). Dunnett’s multiple comparisons test; *p <0.05, **p < 0.01, ***p < 0.001, and ****p < 0.0001 versus WT stress; #p < 0.05 and ^##^p < 0.01 versus siNrf2 stress; n = 5–10.

We then determined whether NIH provide cytoprotection to ARPE-19 from long-term injury. Both WT and siNrf2 ARPE-19 cells were pre-treated, or not, for 24 h with either NIH or DMF, and then exposed to the most challenging stress conditions selected in our previous experiments: 500 µM H_2_O_2_, 100 µM 4-HNE, both for 24 h, or MG132+Baf (5 µM + 50 nM), for 48 h (**Figure 6**).

NIH1, NIH2, and NIH3, significantly protect WT cells from 24 h H_2_O_2_ exposure. In particular, NIH1 displays the best pro-survival effect in stressed cells, by restoring the cell viability to levels not statistically different from control (**Figure 6A**). ARPE-19 cells pre-treated with NIH4 show no change in the cell viability when compared to H_2_O_2_-exposed cells; DMF moderately counteracts the H_2_O_2_-induced mortality, although to a much less extent than any active NIH. In siNrf2 ARPE-19, only NIH1 counteracts the H_2_O_2_-induced mortality, doubling cell survival.

In 24-h 4-HNE-exposed WT cells, NIH1, NIH2, NIH3, as well as DMF, display similar beneficial effects (**Figure 6B**). Analogously to that observed under H_2_O_2_, only the pre-treatment with NIH1 assures a protection from 4-HNE in siNrf2 cells, leading again to a 2.4-fold increase in the cell viability.

In WT cells exposed to MG132+Baf for 48 h, the NIH1, NIH2 and NIH3, as well as DMF, show a significant protective effect, that is abolished in siNrf2 cells (**Figure 6C**).

**Figure 7:**
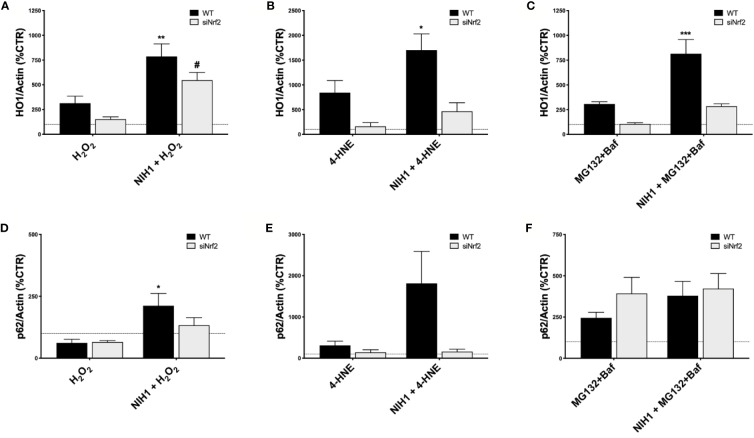
NIH1-mediated modulation of HO1 and p62 protein expression in stressed WT and siNrf2 ARPE-19 cells. Both WT and siNrf2 ARPE-19 cell lines were pre-treated for 24 h with either solvent (DMSO, CTR), or NIH1 [5 μM], and then stressed, or not, for 6 h with H_2_O_2_ [300 μM] **(A**, **D)**, 4-HNE [50 μM] (**B**, **E**), or MG132 + Bafilomycin [5 μM + 50 nM] **(C**, **F)**. Total cellular homogenates were examined by Western blot using antibodies against HO1 **(A**–**C)** and p62 **(D**–**F)**; Actin content was used as a loading control to normalize the data. Results are expressed as percentage means ± SEM in comparison with control (dot line, 100%). Sidak’s multiple comparisons test; *p < 0.05, **p < 0.01; and ***p < 0.001 versus WT stress; ^#^p < 0.05 versus siNrf2 stress; n = 4–6.

Last, focusing on NIH1 and the same conditions used for our previous study of Nrf2/HO1 activation, we evaluated the HO1 and p62 protein levels in both WT and siNrf2 cells, that were pre-treated or not with NIH1 for 24 h, and then exposed for 6 h to either H_2_O_2_ (300 µM), 4-HNE (50 µM), or MG132+Baf (5 µM + 50 nM) (**Figure 7**). When pre-exposed to NIH1, stressed WT cells present higher levels of HO1 than their stressed counterparts, an effect that is index of preventive, NIH1-mediated activation of Nrf2, and that may contribute to the better cell viability profile observed in these cells. Similarly, we found an increment of p62 protein amount in NIH1-pre-treated WT cells. Surprisingly, we found a trend to increase of HO1 protein expression also in NIH1-pre-exposed siNrf2 cells, reaching statistically significance in the H_2_O_2_ group.

Overall these results demonstrate that a pre-treatment with the active NIH protects ARPE-19 cells by different pro-oxidant *noxae*, and suggest that NIH1 may confer resistance upon adverse conditions to both normal and Nrf2-silenced cells.

## Discussion

AMD is a complex disease whose etiology is multifactorial: aging, genetic components, unhealthy environment and behavior, are all risk factors. Though AMD phenotype can dramatically change with progression, a common feature among the dry and wet forms, and the various stages of this pathology, is the degeneration of RPE, primarily responsible, among many other functions, for phagocytosis of photoreceptor outer segments and ROS scavenging (Strauss, 2005). RPE degeneration causes secondarily adverse effects on photoreceptors and choriocapillaris, finally leading to retinal alterations, visual loss and, in the worst cases, irreversible blindness (Ambati and Fowler, 2012). A consistent body of evidence elucidates the importance of dysregulated antioxidant mechanisms and oxidative stress in the development of AMD, and supports the possible association between Nrf2 deficiency and AMD (Datta et al., 2017; Abokyi et al., 2020). Nrf2-pathway is a master regulator of stress response in RPE; beside the well-documented role in the antioxidant cell defense, Nrf2 is also a key component of the transduction machinery to maintain proteostasis, that is altered in AMD (Pajares et al., 2016). Experimental evidence suggests that Nrf2 decreases in aged retina (Batliwala et al., 2017), and its signaling is impaired in aged RPE exposed to an oxidative insult (Sachdeva et al., 2014); KO animals for *Nrf2* or its downstream genes (i.e. *HO1*) develop age-related RPE degeneration and other AMD-like features (Zhao et al., 2011; Felszeghy et al., 2019). These findings strongly suggest that Nrf2-pathway impairment contributes to RPE degeneration in AMD, and that molecules enhancing Nrf2 activity may be of interest for this pathology (Lu et al., 2016).

In the present study on ARPE-19 cells, we show that different types of pro-oxidant *noxae* activate Nrf2, whose importance in the stress defense has been corroborated by siRNA experiments. We found that, in term of viability, siNrf2 cells present a higher susceptibility than WT cells to H_2_O_2_, 4-HNE, MG132+Baf (**Figure 1**), all stresses able to up-regulate Nrf2 in WT, but not in siNrf2 cells (**Figures 2A–C**). Moreover, by focusing on two Nrf2-targets, we observed a different stress-related response in the gene expression of *HO1* and *p62*. In WT cells, HO1 protein is up-regulated upon any stress (**Figures 2D–F**), while p62 is increased following 4-HNE and MG132+Baf, but not H_2_O_2_ (**Figures 2G–I**). The stress-induced raising of HO1 and p62 protein amounts requires the presence of Nrf2, with the exception of p62 in MG132+Baf-treated siNrf2 cells, that show p62 levels even higher than the stressed WT counterpart (**Figures 2D–I**). This may be explained because p62 is degraded by autophagy, which is likely less efficient in siNrf2 cells, and it is further compromised and engulfed upon autophagy inhibition, thus leading to an accumulation of the autophagy marker p62 (Tang et al., 2019).

Keap1/Nrf2/ARE pathway represents a promising pharmacological target to control common pathological features of many chronic diseases characterized by oxidative stress and inflammation (Robledinos-Antón et al., 2019). Accordingly, many studies suggest the potential cytoprotective role of small molecules as Nrf2 activators in retinal tissues and relative pathologies, such as AMD (Batliwala et al., 2017). With this purpose, we tested in ARPE-19 cells some compounds able to induce Nrf2 pathway (Simoni et al., 2017) (**Supplementary Figure 3A**), by comparing their effects with DMF, a Nrf2-activator currently used in clinic for multiple sclerosis (Montes Diaz et al., 2018). We show that, similarly to 10 μM DMF, each active hybrid (NIH1, NIH2, and NIH3), at 5 μM concentration, is able to induce an early activation of Nrf2, accompanied by an up-regulation of its own expression (**Figures 3B–D**). NIH1, NIH2, and NIH3, activate Nrf2, which translocates to the nucleus and escapes from proteasomal degradation, explaining the higher Nrf2 protein levels in comparison with cells exposed to either the solvent or the inactive NIH4. *Nrf2* mRNA levels significantly increase in ARPE-19 cells upon either NIH1 or NIH2, as well as DMF (**Figure 3D**), indicating that, once activated, nuclear Nrf2 also induces its own gene expression in a self-sustaining feedback, in accordance with previous evidence for other Nrf2 activators (Giudice et al., 2010). Downstream, after exposure to either NIH1 or NIH2, we found an early up-regulation of *HO1* gene expression at both mRNA and protein level (**Figures 4A, B**). NIH3 favors HO1 protein expression, although at a longer time. The most consistent increase of HO1 protein [half-life: about 6 h (Lin et al., 2008)] content is detected for these three NIH at 16 h (NIH1: 5.6-fold, NIH2: 4.7-fold, and NIH3: 3.4-fold the control level, respectively). For all, the increase of HO1 protein is persistent, up to 24 h for NIH2 and NIH3, and up to 48 h for NIH1. Moreover, beside increase of *HO1* gene expression, we demonstrate that NIH1 also favors heme oxygenase activity at 6 h in ARPE-19 cells similarly to hemin, a well-known HO1 inducer (**Supplementary Figure 4**).

Concerning DMF, to our knowledge, this is the first literature study of DMF effects on HO1 protein expression in RPE cells. Intriguingly, despite its capability to induce *HO1* mRNA transcription at 3 and 6 h, DMF is less powerful than NIH1 to up-regulate HO1 protein levels at the longer times considered (9, 16 and 24 h). We cannot exclude that this narrow effect may be due to the low concentration of DMF used in our study, and that higher DMF concentration may instead robustly induce HO1 protein expression. 

The catechol motif, present in both NIH1 and NIH2 structures, becomes active electrophilic ortho-quinone on oxidation, which should provide protection in oxidative conditions (Simoni et al., 2017; Serafini et al., 2019). Furthermore, the electrophilic α,β-unsaturated carbonyl group in Michael-type acceptor compounds, such as NIH1, NIH3, as well as DMF, represents an additional source for Nrf2 activation (Basagni et al., 2019). Coherently, in ARPE-19 cells exposed to H_2_O_2_, NIH1, NIH2, and NIH3, display both direct and indirect antioxidant properties, by counteracting the increase of intracellular ROS levels (**Figure 5**). The direct antioxidant capacity of the active NIH is proportional to the number of functional groups within the chemical structure, with NIH1 showing the best profile (**Figure 5A**). The active NIH protect ARPE-19 cells from H_2_O_2_ not only in virtue of their pro-electrophilic chemical structure, but also of the induction of a Nrf2-mediated antioxidant cellular defense. Indeed, following 24 h NIH pre-treatment, H_2_O_2_–exposed WT cells show lower intracellular ROS levels (**Figure 5B**) and mortality (**Figure 6A**) than stressed cells; strikingly, NIH1 is able to fully preserve the cell viability. The active NIH display cytoprotective effects also in WT ARPE-19 upon 4-HNE and MG132+Baf, although to a less extent (**Figures 6B, C**). In our conditions, the DMF-mediated cytoprotective effects are lower than those observed with any active NIH (**Figure 6**). Moreover, contrary to NIH, DMF does not display direct antioxidant properties (**Figure 5A**), albeit it protects ARPE-19 cells from pro-oxidant stress via Nrf2-pathway activation; in agreement, DMF shows no protective effects in siNrf2 cells upon injury (**Figure 6**). In all the stress conditions, the pro-survival effects of each Nrf2 activator require the presence of Nrf2, being no more observable in siNrf2 cells, with the exception of NIH1 (**Figure 6**), that is able to prevent cell death by doubling the number of still viable cells following the exposure to either H_2_O_2_ or 4-HNE. One-day pre-treatment with NIH1 provides WT cells of preventive Nrf2 activation, and thus higher HO1 and p62 protein levels (**Figure 7**), effects that likely predispose the ARPE-19 cells to counteract more efficiently the following stressors and contribute to increase cell survival.

Last, we also discovered that a 24-h pre-treatment with NIH1 significantly up-regulates HO1 protein levels even in H_2_O_2_-stressed siNrf2 cells (**Figure 7A**), an effect that may contribute to their higher viability. The observation that Nrf2 silencing does not abolish the cytoprotective effects of NIH1 pre-treatment suggests that NIH1 may act on a Nrf2-independent pathway and/or on the remaining Nrf2 amount anyhow present in siNrf2 cells. However, further studies will be performed in future to test these hypotheses.

In the context of Keap1/Nrf2/ARE signaling pathway as a druggable target for AMD and other pathologies, various natural and synthetic Nrf2 activators have been recently tested in both *in vitro* and *in vivo* studies to evaluate their protective effects against different pro-oxidant stimuli (Pietrucha-Dutczak et al., 2018; Cui et al., 2019; Shao et al., 2019; Zhou et al., 2019; Fresta et al., 2020). In this panorama, our NIHs look as noteworthy pharmacological molecules, displaying a good profile of tolerability (**Figure 3**) and uncommon cytoprotective effects in RPE cells under three different types of AMD-related oxidative stress.

In conclusion, our study corroborates the relevance of Nrf2 in the RPE stress response, and shows that, beside WT, siNrf2 ARPE-19 cells exposed to different stress may be a useful *in vitro* model to test pharmacologically active molecules potentially interesting in AMD. Our findings also suggest that the active NIH are potentially valuable protective, preventive tools for cells physiologically facing challenging, high pro-oxidant stress, as RPE. In particular, NIH1 is worthy of further studies on RPE, AMD and other retinal degenerative diseases, and may be useful to reinforce the endogenous cellular protective mechanisms in both normal and Nrf2 impaired conditions.

## Data Availability Statement

All relevant data is contained within the article. The original contributions presented in the study are included in the article/**Supplementary Material**; further inquiries can be directed to the corresponding author.

## Author Contributions

Conceptualization: MA. Methodology: MC, CL, MR, MA. Immunocytochemistry: MC, CL, MA. Chemical synthesis and hybrid stability assay: FB. All other cellular and molecular biology experiments: MC and MA. Data analyses: MC, MA. Critical discussion: MC, CL, MR, SG, MA. Writing of the original draft: MA. Review and editing: MC, CL, MR, SG, MA. Supervision, project administration and funding acquisition: MA.

## Funding

This work was supported by the University of Pavia [to MA, grant number BSR1744747; 2017] and the Italian Ministry of University and Research [to MA, FFABR2017]. The University of Bologna is acknowledged by MR [Grants from RFO].

## Conflict of Interest

The authors declare that the research was conducted in the absence of any commercial or financial relationships that could be construed as a potential conflict of interest.

## References

[B1] Abokyi S. To C.-H. Lam T. T. Tse D. Y. (2020). Central Role of Oxidative Stress in Age-Related Macular Degeneration: Evidence from a Review of the Molecular Mechanisms and Animal Models. Oxid. Med. Cell. Longev. 2020, 1–19. 10.1155/2020/7901270 PMC7035553 32104539

[B2] Amadio M. Scapagnini G. Laforenza U. Intrieri M. Romeo L. Govoni S. (2008). Post-transcriptional regulation of HSP70 expression following oxidative stress in SH-SY5Y cells: the potential involvement of the RNA-binding protein HuR. Curr. Pharm. Des. 14, 2651–2658. 10.2174/138161208786264052 18991684

[B3] Amadio M. Scapagnini G. Davinelli S. Calabrese V. Govoni S. Pascale A. (2014). Involvement of ELAV RNA-binding proteins in the post-transcriptional regulation of HO-1. Front. Cell. Neurosci. 8, 459. 10.3389/fncel.2014.00459 25642166 PMC4295526

[B4] Amadio M. Govoni S. Pascale A. (2016). Targeting VEGF in eye neovascularization: What’s new? Pharmacol. Res. 103, 253–269. 10.1016/j.phrs.2015.11.027 26678602

[B5] Ambati J. Fowler B. J. (2012). Mechanisms of Age-Related Macular Degeneration. Neuron 75, 26–39. 10.1016/j.neuron.2012.06.018 22794258 PMC3404137

[B6] Basagni F. Lanni C. Minarini A. Rosini M. (2019). Lights and shadows of electrophile signaling: focus on the Nrf2-Keap1 pathway. Future Med. Chem. 11, 707–721. 10.4155/fmc-2018-0423 30942112

[B7] Batliwala S. Xavier C. Liu Y. Wu H. Pang I.-H. (2017). Involvement of Nrf2 in Ocular Diseases. Oxid. Med. Cell. Longev. 2017, 1703810. 10.1155/2017/1703810 28473877 PMC5394909

[B8] Bhutto I. Lutty G. (2012). Understanding age-related macular degeneration (AMD): relationships between the photoreceptor/retinal pigment epithelium/Bruch’s membrane/choriocapillaris complex. Mol. Asp. Med. 33, 295–317. 10.1016/j.mam.2012.04.005 PMC3392421 22542780

[B9] Chen J. Wang L. Chen Y. Sternberg P. Cai J. (2009). Phosphatidylinositol 3 Kinase Pathway and 4-Hydroxy-2-Nonenal-Induced Oxidative Injury in the RPE. Investig. Opthalmol. Vis. Sci. 50, 936. 10.1167/iovs.08-2439 PMC2716057 18806289

[B10] Cui R. Tian L. Lu D. Li H. Cui J. (2019). Exendin-4 Protects Human Retinal Pigment Epithelial Cells from H2O2-Induced Oxidative Damage via Activation of NRF2 Signaling. Ophthal. Res., 2019. 1–9. 10.1159/000504891 31865348

[B11] Datta S. Cano M. Ebrahimi K. Wang L. Handa J. T. (2017). The impact of oxidative stress and inflammation on RPE degeneration in non-neovascular AMD. Prog. Retin. Eye Res. 60, 201–218. 10.1016/j.preteyeres.2017.03.002 28336424 PMC5600827

[B12] Ethen C. M. Reilly C. Feng X. Olsen T. W. Ferrington D. A. (2007). Age-related macular degeneration and retinal protein modification by 4-hydroxy-2-nonenal. Invest. Ophthalmol. Vis. Sci. 48, 3469–3479. 10.1167/iovs.06-1058 17652714

[B13] Felszeghy S. Viiri J. Paterno J. J. Hyttinen J. M. T. Koskela A. Chen M. (2019). Loss of NRF-2 and PGC-1α genes leads to retinal pigment epithelium damage resembling dry age-related macular degeneration. Redox Biol. 20, 1–12. 10.1016/j.redox.2018.09.011 30253279 PMC6156745

[B14] Ferrington D. A. Sinha D. Kaarniranta K. (2016). Defects in retinal pigment epithelial cell proteolysis and the pathology associated with age-related macular degeneration. Prog. Retin. Eye Res. 51, 69–89. 10.1016/j.preteyeres.2015.09.002 26344735 PMC4769684

[B15] Foresti R. Bucolo C. Platania C. M. B. Drago F. Dubois-Randé J.-L. Motterlini R. (2015). Nrf2 activators modulate oxidative stress responses and bioenergetic profiles of human retinal epithelial cells cultured in normal or high glucose conditions. Pharmacol. Res. 99, 296–307. 10.1016/j.phrs.2015.07.006 26188148

[B16] Fresta C. G. Fidilio A. Lazzarino G. Musso N. Grasso M. Merlo S. (2020). Modulation of Pro-Oxidant and Pro-Inflammatory Activities of M1 Macrophages by the Natural Dipeptide Carnosine. Int. J. Mol. Sci. 21 (3), 776. 10.3390/ijms21030776 PMC7038063 31991717

[B17] Giudice A. Arra C. Turco M. C. (2010). ““Review of Molecular Mechanisms Involved in the Activation of the Nrf2-ARE Signaling Pathway by Chemopreventive Agents,” in Transcription Factors. Ed. Higgins P. J. (Totowa, NJ: Humana Press), 37–74. 10.1007/978-1-60761-738-9_3 20694660

[B18] Golestaneh N. Chu Y. Xiao Y.-Y. Stoleru G. L. Theos A. C. (2018). Dysfunctional autophagy in RPE, a contributing factor in age-related macular degeneration. Cell Death Dis. 8, e2537–e2537. 10.1038/cddis.2016.453 PMC5386365 28055007

[B19] Hu X. Liang Y. Zhao B. Wang Y. (2019). Thymoquinone protects human retinal pigment epithelial cells against hydrogen peroxide induced oxidative stress and apoptosis. J. Cell. Biochem. 120, 4514–4522. 10.1002/jcb.27739 30269355

[B20] Hyttinen J. M. T. Amadio M. Viiri J. Pascale A. Salminen A. Kaarniranta K. (2014). Clearance of misfolded and aggregated proteins by aggrephagy and implications for aggregation diseases. Ageing Res. Rev. 18, 16–28. 10.1016/j.arr.2014.07.002 25062811

[B21] Jager R. D. Mieler W. F. Miller J. W. (2008). Age-related macular degeneration. N. Engl. J. Med. 358, 2606–2617. 10.1056/NEJMra0801537 18550876

[B22] Jain A. Lamark T. Sjøttem E. Larsen K. B. Awuh J. A. Øvervatn A. (2010). p62/SQSTM1 is a target gene for transcription factor NRF2 and creates a positive feedback loop by inducing antioxidant response element-driven gene transcription. J. Biol. Chem. 285, 22576–22591. 10.1074/jbc.M110.118976 20452972 PMC2903417

[B23] Jiang T. Harder B. Rojo de la Vega M. Wong P. K. Chapman E. Zhang D. D. (2015). p62 links autophagy and Nrf2 signaling. Free Radic. Biol. Med. 88, 199–204. 10.1016/j.freeradbiomed.2015.06.014 26117325 PMC4628872

[B24] Kaarniranta K. Ryhänen T. Karjalainen H. M. Lammi M. J. Suuronen T. Huhtala A. (2005). Geldanamycin increases 4-hydroxynonenal (HNE)-induced cell death in human retinal pigment epithelial cells. Neurosci. Lett. 382, 185–190. 10.1016/j.neulet.2005.03.009 15911146

[B25] Kaarniranta K. Tokarz P. Koskela A. Paterno J. Blasiak J. (2017). Autophagy regulates death of retinal pigment epithelium cells in age-related macular degeneration. Cell Biol. Toxicol. 33, 113–128. 10.1007/s10565-016-9371-8 27900566 PMC5325845

[B26] Kaemmerer E. Schutt F. Krohne T. U. Holz F. G. Kopitz J. (2007). Effects of Lipid Peroxidation-Related Protein Modifications on RPE Lysosomal Functions and POS Phagocytosis. Investig. Opthalmol. Vis. Sci. 48, 1342. 10.1167/iovs.06-0549 17325182

[B27] Lambros M. L. Plafker S. M. (2016). “Oxidative Stress and the Nrf2 Anti-Oxidant Transcription Factor in Age-Related Macular Degeneration,” in Retinal Degenerative Diseases. Eds. Bowes Rickman C. LaVail M. M. Anderson R. E. Grimm C. Hollyfield J. Ash J. (Cham: Springer International Publishing), 67–72. 10.1007/978-3-319-17121-0_10 PMC5757825 26427395

[B28] Lim L. S. Mitchell P. Seddon J. M. Holz F. G. Wong T. Y. (2012). Age-related macular degeneration. Lancet 379, 1728–1738. 10.1016/S0140-6736(12)60282-7 22559899

[B29] Lin P.-H. Chiang M.-T. Chau L.-Y. (2008). Ubiquitin-proteasome system mediates heme oxygenase-1 degradation through endoplasmic reticulum-associated degradation pathway. Biochim. Biophys. Acta 1783, 1826–1834. 10.1016/j.bbamcr.2008.05.008 18544348

[B30] Loboda A. Damulewicz M. Pyza E. Jozkowicz A. Dulak J. (2016). Role of Nrf2/HO-1 system in development, oxidative stress response and diseases: an evolutionarily conserved mechanism. Cell. Mol. Life Sci. CMLS 73, 3221–3247. 10.1007/s00018-016-2223-0 27100828 PMC4967105

[B31] Lu M.-C. Ji J.-A. Jiang Z.-Y. You Q.-D. (2016). The Keap1-Nrf2-ARE Pathway As a Potential Preventive and Therapeutic Target: An Update: THE KEAP1-NRF2-ARE PATHWAY. Med. Res. Rev. 36, 924–963. 10.1002/med.21396 27192495

[B32] Marchesi N. Thongon N. Pascale A. Provenzani A. Koskela A. Korhonen E. (2018). Autophagy Stimulus Promotes Early HuR Protein Activation and p62/SQSTM1 Protein Synthesis in ARPE-19 Cells by Triggering Erk1/2, p38 ^MAPK^ , and JNK Kinase Pathways. Oxid. Med. Cell. Longev. 2018, 1–15. 10.1155/2018/4956080 PMC5822911 29576851

[B33] McMahon M. Itoh K. Yamamoto M. Hayes J. D. (2003). Keap1-dependent proteasomal degradation of transcription factor Nrf2 contributes to the negative regulation of antioxidant response element-driven gene expression. J. Biol. Chem. 278, 21592–21600. 10.1074/jbc.M300931200 12682069

[B34] Montes Diaz G. Hupperts R. Fraussen J. Somers V. (2018). Dimethyl fumarate treatment in multiple sclerosis: Recent advances in clinical and immunological studies. Autoimmun. Rev. 17, 1240–1250. 10.1016/j.autrev.2018.07.001 30316988

[B35] Motohashi H. Yamamoto M. (2004). Nrf2-Keap1 defines a physiologically important stress response mechanism. Trends Mol. Med. 10, 549–557. 10.1016/j.molmed.2004.09.003 15519281

[B36] Pajares M. Jiménez-Moreno N. García-Yagüe Á. J. Escoll M. de Ceballos M. L. Van Leuven F. (2016). Transcription factor NFE2L2/NRF2 is a regulator of macroautophagy genes. Autophagy 12, 1902–1916. 10.1080/15548627.2016.1208889 27427974 PMC5079676

[B37] Pietrucha-Dutczak M. Amadio M. Govoni S. Lewin-Kowalik J. Smedowski A (2018). The Role of Endogenous Neuroprotective Mechanisms in the Prevention of Retinal Ganglion Cells Degeneration. Front. Neurosci. 12, 834. 10.3389/fnins.2018.00834 30524222 PMC6262299

[B38] Robledinos-Antón N. Fernández-Ginés R. Manda G. Cuadrado A. (2019). Activators and Inhibitors of NRF2: A Review of Their Potential for Clinical Development. Oxid. Med. Cell. Longev. 2019, 9372182. 10.1155/2019/9372182 31396308 PMC6664516

[B39] Sachdeva M. M. Cano M. Handa J. T. (2014). Nrf2 signaling is impaired in the aging RPE given an oxidative insult. Exp. Eye Res. 119, 111–114. 10.1016/j.exer.2013.10.024 24216314 PMC3946784

[B40] Serafini M. M. Catanzaro M. Fagiani F. Simoni E. Caporaso R. Dacrema M. (2019). Modulation of Keap1/Nrf2/ARE Signaling Pathway by Curcuma- and Garlic-Derived Hybrids. Front. Pharmacol. 10, 1597. 10.3389/fphar.2019.01597 32047434 PMC6997134

[B41] Shao Y. Yu H. Yang Y. Li M. Hang L. Xu X. (2019). A Solid Dispersion of Quercetin Shows Enhanced Nrf2 Activation and Protective Effects against Oxidative Injury in a Mouse Model of Dry Age-Related Macular Degeneration. Oxid. Med. Cell. Longev. 2019, 1479571. 10.1155/2019/1479571 31781321 PMC6875405

[B42] Simoni E. Serafini M. M. Bartolini M. Caporaso R. Pinto A. Necchi D. (2016). Nature-Inspired Multifunctional Ligands: Focusing on Amyloid-Based Molecular Mechanisms of Alzheimer’s Disease. ChemMedChem 11, 1309–1317. 10.1002/cmdc.201500422 26497622

[B43] Simoni E. Serafini M. M. Caporaso R. Marchetti C. Racchi M. Minarini A. (2017). Targeting the Nrf2/Amyloid-Beta Liaison in Alzheimer’s Disease: A Rational Approach. ACS Chem. Neurosci. 8, 1618–1627. 10.1021/acschemneuro.7b00100 28421738

[B44] Strauss O. (2005). The retinal pigment epithelium in visual function. Physiol. Rev. 85, 845–881. 10.1152/physrev.00021.2004 15987797

[B45] Tang Z. Hu B. Zang F. Wang J. Zhang X. Chen H. (2019). Nrf2 drives oxidative stress-induced autophagy in nucleus pulposus cells via a Keap1/Nrf2/p62 feedback loop to protect intervertebral disc from degeneration. Cell Death Dis. 10, 510. 10.1038/s41419-019-1701-3 31263165 PMC6602960

[B46] Viiri J. Hyttinen J. M. T. Ryhänen T. Rilla K. Paimela T. Kuusisto E. (2010). p62/sequestosome 1 as a regulator of proteasome inhibitor-induced autophagy in human retinal pigment epithelial cells. Mol. Vis. 16, 1399–1414. 20680098 PMC2913138

[B47] Viiri J. Amadio M. Marchesi N. Hyttinen J. M. T. Kivinen N. Sironen R. (2013). Autophagy activation clears ELAVL1/HuR-mediated accumulation of SQSTM1/p62 during proteasomal inhibition in human retinal pigment epithelial cells. PloS One 8, e69563. 10.1371/journal.pone.0069563 23922739 PMC3726683

[B48] Wang L. Ebrahimi K. B. Chyn M. Cano M. Handa J. T. Ash J. (2016). “Biology of p62/sequestosome-1 in Age-Related Macular Degeneration (AMD),” in Retinal Degenerative Diseases. Eds. Bowes Rickman C. LaVail M. M. Anderson R. E. Grimm C. Hollyfield J. (Cham: Springer International Publishing)), 17–22. 10.1007/978-3-319-17121-0_3

[B49] Zhang H. Davies K. J. A. Forman H. J. (2015). Oxidative stress response and Nrf2 signaling in aging. Free Radic. Biol. Med. 88, 314–336. 10.1016/j.freeradbiomed.2015.05.036 26066302 PMC4628850

[B50] Zhao Z. Chen Y. Wang J. Sternberg P. Freeman M. L. Grossniklaus H. E. (2011). Age-Related Retinopathy in NRF2-Deficient Mice. PloS One 6, e19456. 10.1371/journal.pone.0019456 21559389 PMC3084871

[B51] Zhao J. Tan S. Liu F. Zhang Y. Su M. Sun D. (2012). Heme oxygenase and ocular disease: a review of the literature. Curr. Eye Res. 37, 955–960. 10.3109/02713683.2012.700753 22720721

[B52] Zhao H. Wang R. Ye M. Zhang L. (2019). Genipin protects against H2O2-induced oxidative damage in retinal pigment epithelial cells by promoting Nrf2 signaling. Int. J. Mol. Med. 43, 936–944. 10.3892/ijmm.2018.4027 30569096 PMC6317649

[B53] Zhou Y. Zhou L. Zhou K. Zhang J. Shang F. Zhang X. (2019). Celastrol Protects RPE Cells from Oxidative Stress-Induced Cell Death via Activation of Nrf2 Signaling Pathway. Curr. Mol. Med. 19, 172–182. 10.2174/1566524019666190424131704 31032752

[B54] Zhu C. Dong Y. Liu H. Ren H. Cui Z. (2017). Hesperetin protects against H 2 O 2 -triggered oxidative damage via upregulation of the Keap1-Nrf2/HO-1 signal pathway in ARPE-19 cells. Biomed. Pharmacother. 88, 124–133. 10.1016/j.biopha.2016.11.089 28103505

