# Supplementary figures and images for: Eye-Light on Age-Related Macular Degeneration: Targeting Nrf2-Pathway as a Novel Therapeutic Strategy for Retinal Pigment Epithelium
Source: Front Pharmacol. 2020 Jun 5;11:844. doi: 10.3389/fphar.2020.00844 (PMC7291861; doi:10.3389/fphar.2020.00844)

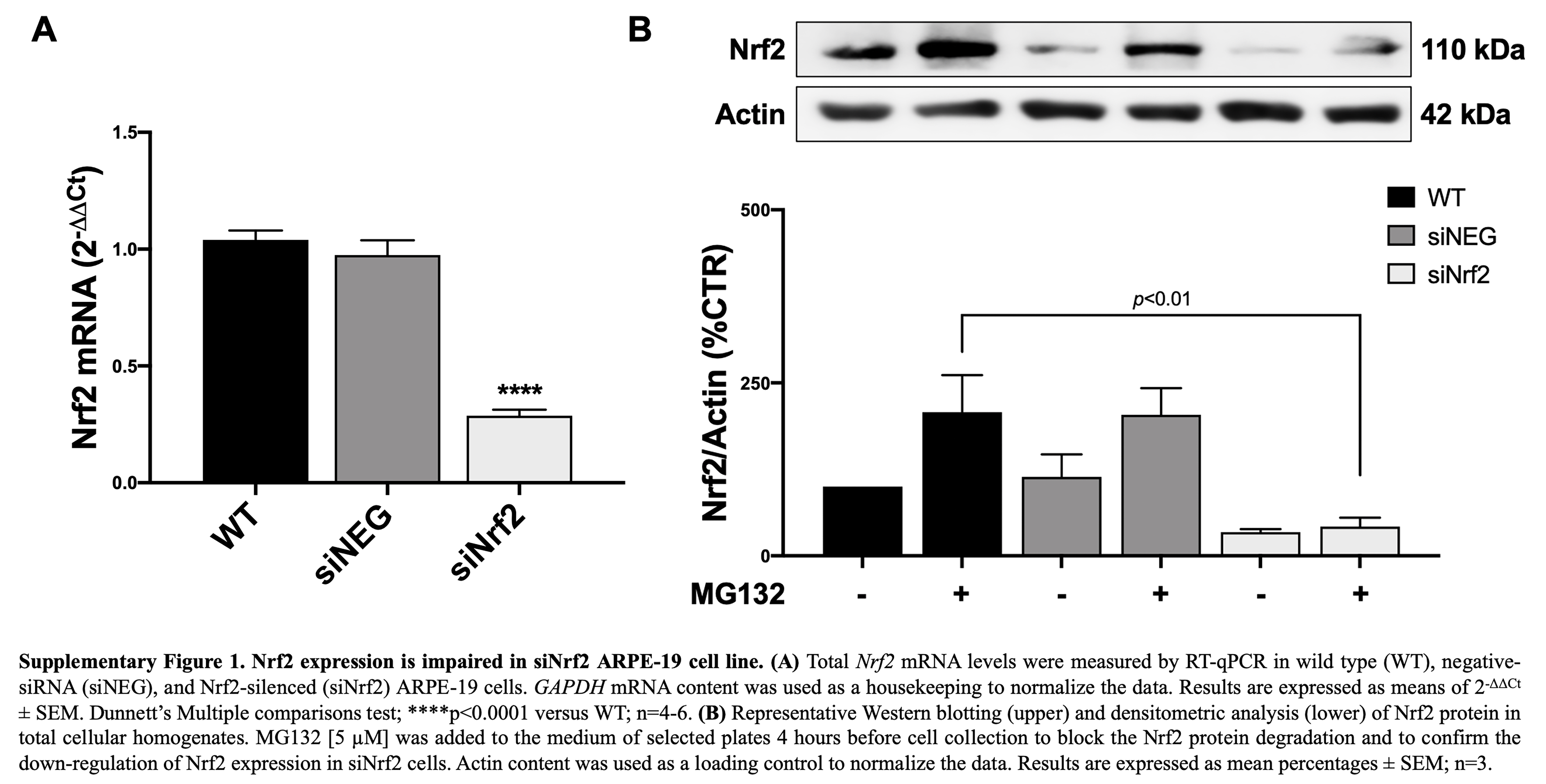

Supplement: Supplementary file 1 [file Image_1.jpeg]

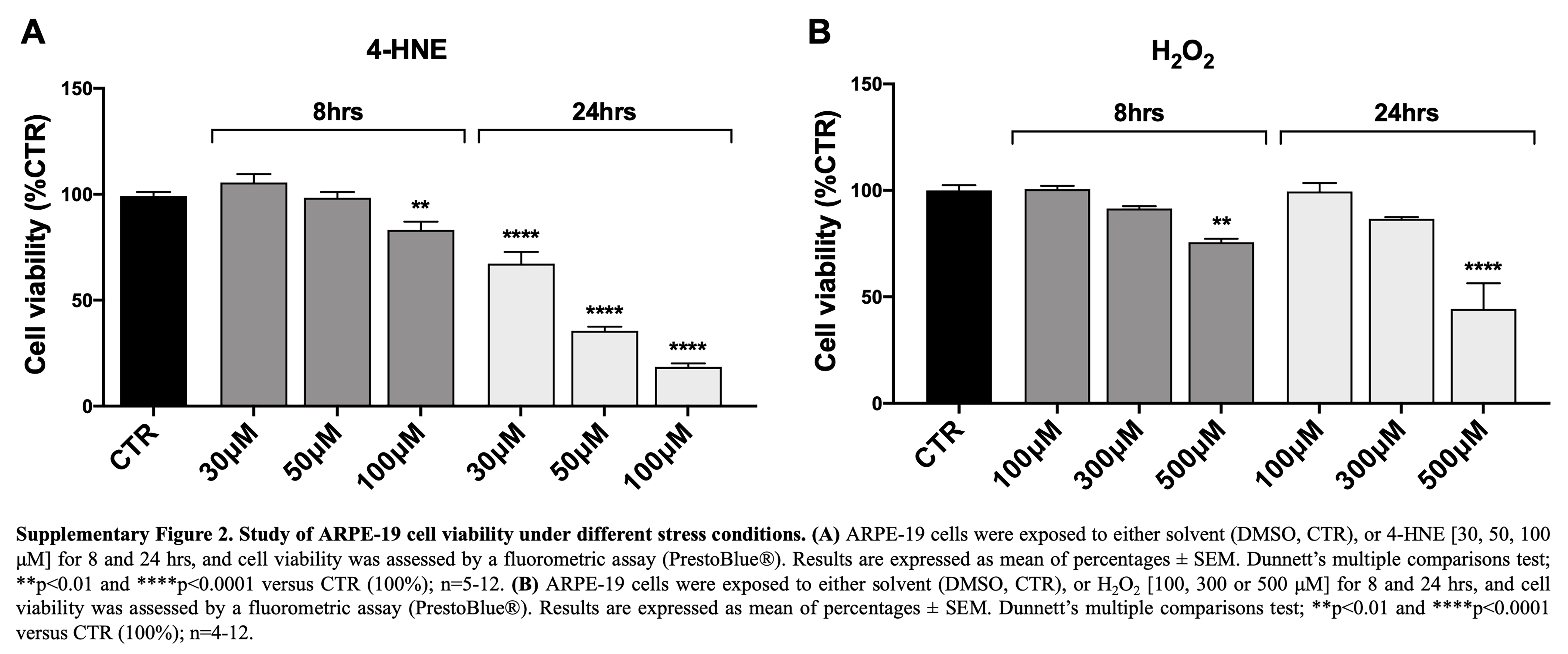

Supplement: Supplementary file 2 [file Image_2.jpeg]

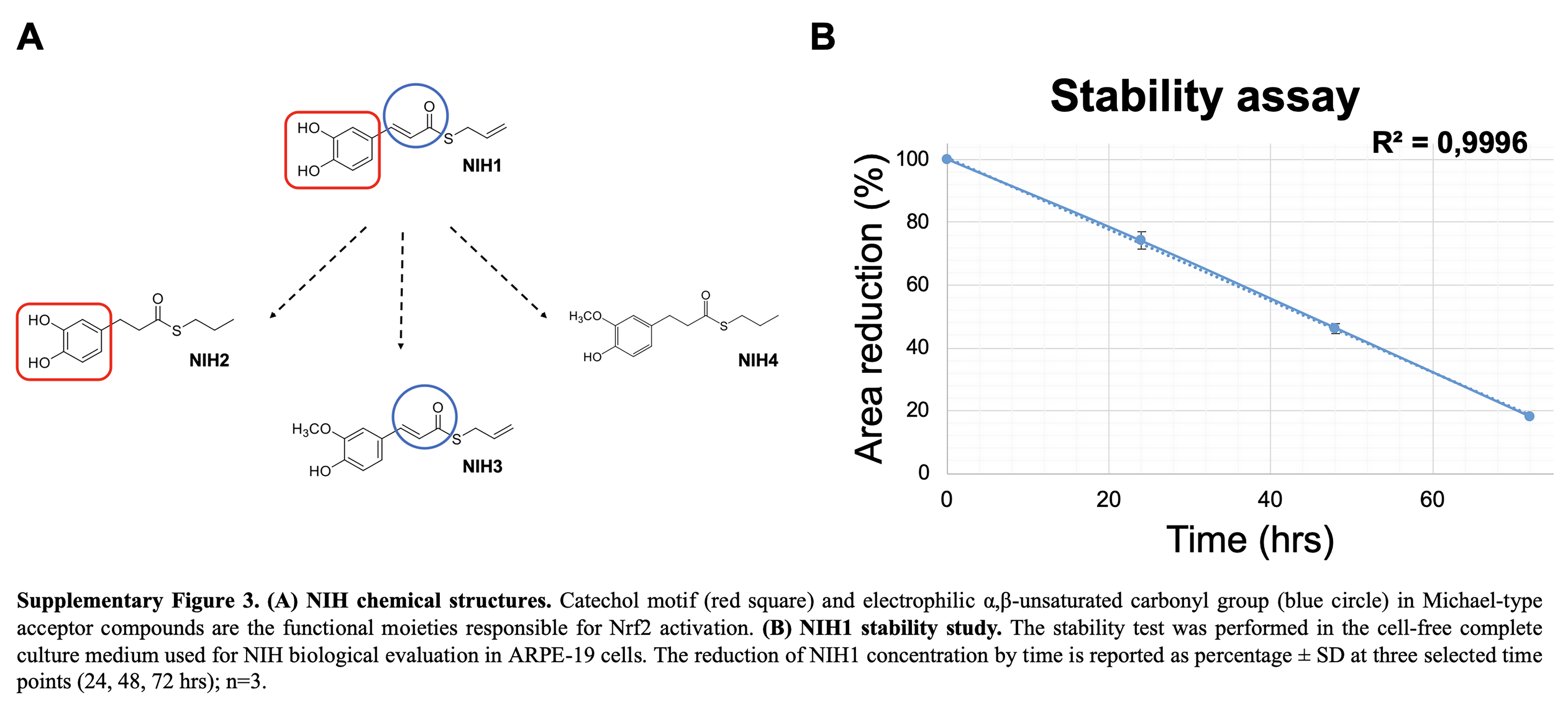

Supplement: Supplementary file 3 [file Image_3.jpeg]

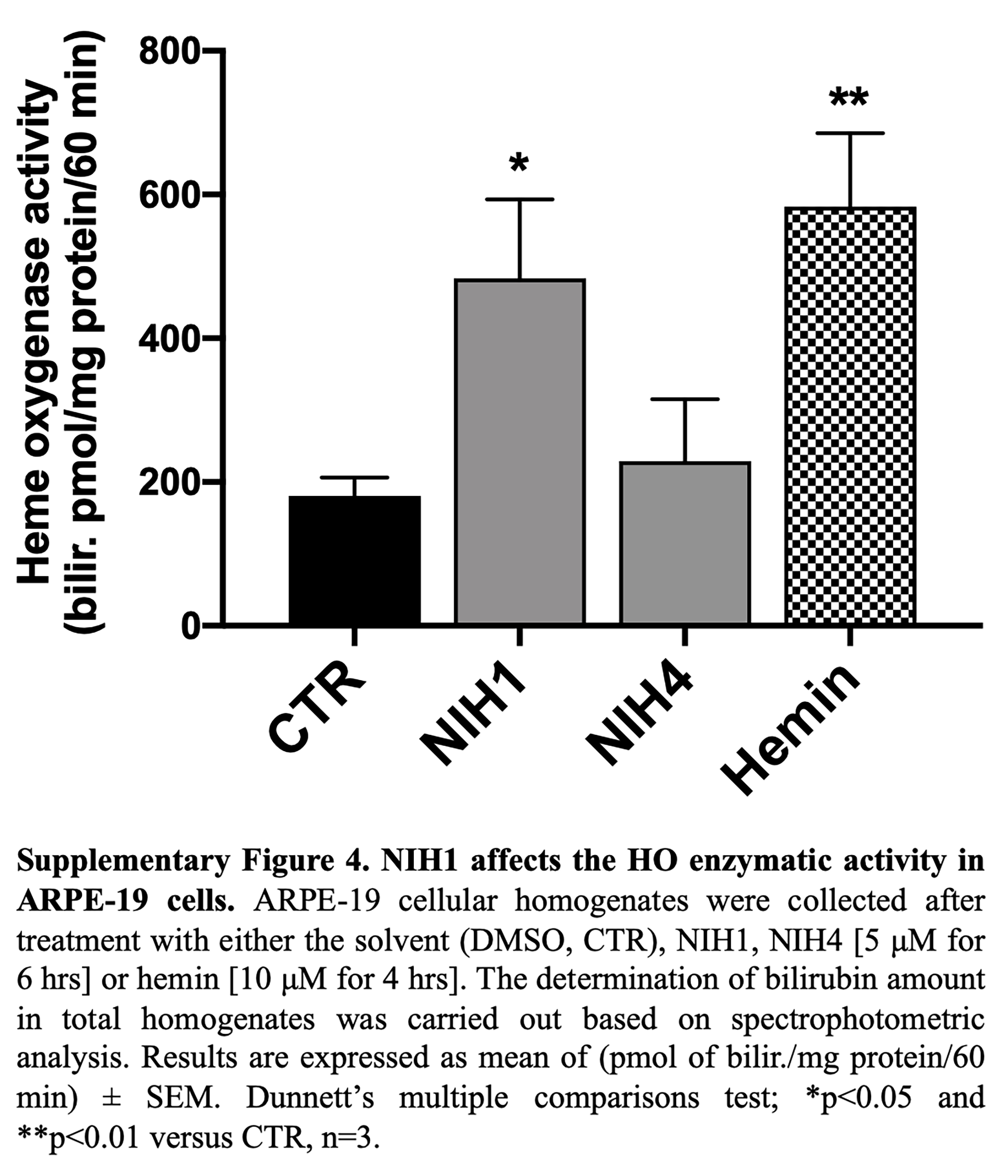

Supplement: Supplementary file 4 [file Image_4.jpeg]
